# Multiscale variability in coral recruitment in the Mascarene Islands: From centimetric to geographical scale

**DOI:** 10.1371/journal.pone.0214163

**Published:** 2019-03-22

**Authors:** Florian Jouval, Anne Catherine Latreille, Sophie Bureau, Mehdi Adjeroud, Lucie Penin

**Affiliations:** 1 UMR 9220 ENTROPIE, Université de La Réunion, Faculté des Sciences et Technologies & Laboratoire d’Excellence CORAIL, La Réunion, France; 2 UMR 249 PIMIT, Université de La Réunion, INSERM, CNRS, IRD, Plateforme Technologique CYROI, La Réunion, France; 3 UMR 9220 ENTROPIE, Institut de Recherche pour le Développement (IRD) & Laboratoire d’Excellence CORAIL, Université de Perpignan Via Domitia, Perpignan, France; Department of Agriculture and Water Resources, AUSTRALIA

## Abstract

Coral recruitment refers to the processes allowing maintenance and renewal of coral communities. Recruitment success is therefore indispensable for coral reef recovery after disturbances. Recruitment processes are governed by a variety of factors occurring at all spatial and temporal scales, from centimetres to hundreds of kilometres. In the present context of rising disturbances, it is thus of major importance to better understand the relative importance of different scales in this variation, and when possible, the factors associated with these scales. Multiscale spatio-temporal variability of scleractinian coral recruitment was investigated at two of the Mascarene Islands: Reunion and Rodrigues. Recruitment rates and taxonomic composition were examined during three consecutive six-month periods from regional to micro-local scales (i.e. from hundreds of kilometres to few centimetres) and between two protection levels (no-take zones and general protection zones). Very low recruitment rates were observed. Rodrigues displayed lower recruitment rates than Reunion. Recruit assemblage was dominated by Pocilloporidae (77.9%), followed by Acroporidae (9.9%) and Poritidae (5.2%). No protection effect was identified on coral recruitment, despite differences in recruitment rates among sites within islands. Recruits were patchily distributed within sites but no aggregative effect was detected, i.e. the preferentially colonised tiles were not spatially grouped. Recruits settled mainly on the sides of the tiles, especially at Rodrigues, which could be attributed to the high concentration of suspended matter. The variability of recruitment patterns at various spatial scales emphasises the importance of micro- to macro-local variations of the environment in the dynamics and maintenance of coral populations. High temporal variability was also detected, between seasons and years, which may be related to the early 2016 bleaching event at Rodrigues. The low recruitment rates and the absence of protection effect raise questions about the potential for recovery from disturbances of coral reefs in the Mascarene Islands.

## Introduction

Coral recruitment processes, i.e. settlement of larvae and early post-settlement events occurring in the first weeks and months of benthic life largely shape coral reef community structure [1], allowing maintenance and renewal of coral communities through replacement of dead adults [2,3]. An essential component of reef resilience, recruitment largely influences reef recovery following mass mortalities induced by large-scale disturbances [1,4,5]. Coral recruitment success is thus necessary for coral reef recovery after disturbances, and recruitment failure is a known factor of phase shift [2,6].

Recruitment processes are governed by a variety of factors occurring at all spatial and temporal scales, from centimetres to hundreds of kilometres, and from seasons to decades. This creates spatio-temporal variability in measures of recruitment descriptors at all scales that have been investigated so far [7–11]. Among factors that can be cited, recruitment depends upon spawning of adults, that is generally initiated by increasing sea temperature [12], moon phase [13] and wind fields [14]. Fertilization success and embryo survival are influenced by local environmental factors [15,16]. Larval dispersal is mainly driven by larval survival and pelagic larval duration, associated with oceanographic conditions experienced by larvae, like tidal or wind-driven currents [17]. Larval settlement and survival depend on local (metric scale) and micro-local (centimetric scale) parameters such as the presence of biological inducers or competitors [18–21], light [22], sedimentation [23] or herbivorous grazing [24]. At large geographic scales, the biological and ecological processes that sustain coral communities may be affected by disturbances such as cyclones, bleaching events or outbreaks of the coral predator *Acanthaster planci* [25,26]. Moreover, anthropogenic impacts interfere with recruitment processes at the local scale. For example, stressors such as pollution, eutrophication, sedimentation or overfishing have been linked to deleterious effects on coral recruitment [11,27–29]. In the present context of rising disturbance frequency and intensity, coral reefs will have to recover more often than in previous decades [30–32]. It is thus of capital importance to better understand the relative importance of different scales in the spatial and temporal variation of recruitment, and where possible, the factors associated with these variations.

A growing body of literature describes spatio-temporal variability of coral recruitment. Even if long-term studies are scarce (but see [33]), important year to year variation has been documented [1,33,34]. Seasonal variation is also well established, with summer periods typically more favourable for coral settlement [e.g. 8,34,35]. Regarding spatial variability, regional variation appears to be important; six-fold differences were observed among reef regions on the Great Barrier Reef (GBR) [1], and five-fold differences reported among islands of the Society Archipelago (French Polynesia [36]). Within a region or an island, at the scale of a few kilometres, some reefs or sites also typically receive more recruits than others, and these large-scale patterns seem to be consistent in time, among seasons and years [1,34,36,37]. At a much smaller scale, settlement and recruitment vary among micro-habitats as described by differences in recruitment rates among tile orientations [34,37,38]. Between these two scales, local variation patterns, i.e. within-site variation, at the scale of the metre, and its consistency in time has rarely been investigated (but see [39]). This overlooked scale of variation deserves better investigation since many biotic and abiotic factors like competition, predation, light or hydrodynamic conditions vary greatly at this scale (e.g. [24,29,38,40]).

In recent decades, management tools, such as marine protected areas (MPAs), have been developed around the world to limit the anthropogenic impacts on the marine environment. In general, these MPAs delimit different areas in which certain activities, and fishing in particular, are allowed or prohibited [41]. While fishing bans generally improve fish biomass and diversity when enforced [42,43], their effects on coral abundance, while generally positive, are more tenuous [44,45]. Mechanisms involved in positive effects on corals include lower damage from fishing gear [46] and higher rates of herbivory, tipping the coral-algal competition balance towards coral success [43]. Increases in coral recruitment rates can be expected in this context of lower competition with turf and macroalgae, which are known to limit settlement and survivorship [29,47–49]. In contrast, higher herbivorous fish biomass in MPAs can be linked with higher incidental predation of early stage corals by herbivores [50], but see Mumby [51]. While some studies have compared recruitment rates inside and outside MPAs (e.g. [10,11,52,53]), few have concluded a positive effect of MPAs on coral recruitment (but see [54]).

The present study aims to evaluate (i) the temporal variability of coral recruitment at interannual and seasonal scales, as well as the spatial variation of coral recruitment between two islands of the Mascarene Archipelago (hundreds of kms), among sites of each island (km), among tiles within sites (m), and within settlement tiles (cm); as well as (ii) the effects of MPA status (fishing ban) on coral recruitment. Recruitment rates and taxonomic composition were thus examined during three consecutive six-month periods at regional (between two islands, Reunion and Rodrigues), island (between four sites at each island), local (between 20 artificial settlement tiles at each site) and micro-local levels through the orientation of recruits on artificial settlement tiles. The variation of recruitment rates and composition were also examined within and outside no-take zones (NTZs) at both Reunion and Rodrigues reefs.

## Material and methods

### Ethics statements

Fieldwork was conducted within the Réserve Naturelle Marine de La Réunion (MPA) at Reunion, under research authorization N°2014–27 DEAL/SEB/UBIO, and within South East Marine Protected Area at Rodrigues, under Rodrigues Regional Assembly research authorization N°RA 402/17 Vol II.

### Study sites

**Fig 1:**
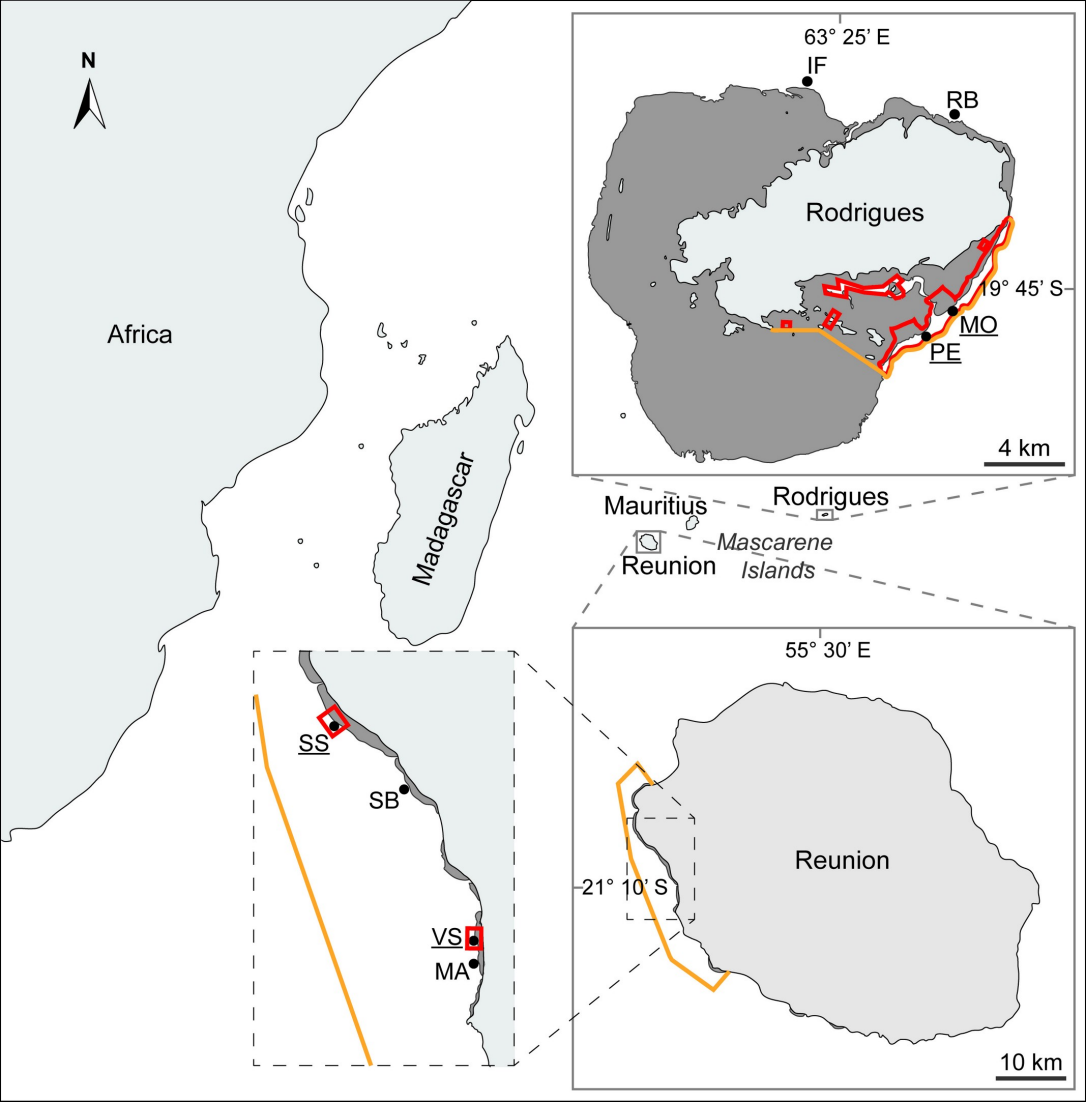
Location of the sampling sites (black dots) on Reunion and Rodrigues reefs. Light grey: land; dark grey: reef flat; orange: MPAs boundaries; red: NTZs boundaries. Underlined sampling sites belong to NTZs. SS: Sanctuaire Sud; SB: Souris Blanche; VS: Varangue Sud; MA: Marine; IF: Ile aux Fous; RB: Rivière Banane; MO: Mourouk; PE: Port Sud-Est.

Reunion and Rodrigues islands are part of the Mascarene Islands, along with Mauritius, in the South-Western Indian Ocean (SWIO). Reunion (21° 07’ S, 55° 32’ E), one of the overseas French territories, is a volcanic island (*ca*. 70 km long and 50 km wide) located 700 km east of Madagascar. Fringing reefs line the island in a 12 km^2^ area along 25 km of coastline on its west and south coasts [55]. Rodrigues (19° 43’ S, 63° 25’ E) is a small isolated island that is part of the Republic of Mauritius (18.3 km long and 6.5 km wide), and is the easternmost of the Mascarene Islands. It is surrounded by an almost continuous 90 km long reef rim and constitutes a reef area of *ca*. 200 km^2^ [56]. Deleterious effects of coral bleaching are growing stronger over the decades in the Mascarene Islands (see [57] at Rodrigues). Reunion and Rodrigues reefs are not immune to other anthropogenic stress either. Increased urbanisation of the Reunion coastline during the last decades drove noticeable eutrophication in the reef environment [58] and a decrease in reef fish stocks in some areas [59]. These phenomena have led to an increase in the relative cover of algae compared to corals [58,60,61]. Consequently, a multiple-use MPA (Réserve Naturelle Marine de La Réunion) was set up in 2007 to address the deterioration of reefs. This MPA, covering an area of over 35 km^2^, is divided into three levels of protection: open areas for human activities (general protection zones or GPZs), restricted areas where only some traditional and commercial fishing activities are allowed, and sanctuaries where no activity is allowed, thus corresponding to NTZs (Fig 1). Human pressure is less documented for the Rodrigues reef. In response to overfishing 10 years ago, the local government of Rodrigues implemented four MPAs to the north of the island, but only partial management has been set up and fishing still occurs in these areas [62,63]. In addition, the South East Marine Protected Area (SEMPA) is an area in the south-eastern of Rodrigues designated as an MPA in 2009. It includes both inner and outer lagoon covering a total 43.7 km^2^ divided into different multiple-use zones, including NTZs where fishing is prohibited (Fig 1).

### Sampling strategy

The sampling strategy used four sites on the outer reef slopes at both Reunion and Rodrigues islands, including two sites within a NTZ and two sites outside NTZ for each island (Fig 1). Eight sites were thus sampled and coded as follows: Reunion (SS: Sanctuaire Sud and VS: Varangue Sud in the NTZ; SB: Souris Blanche and MA: Marine in GPZs); Rodrigues (MO: Mourouk and PE: Port Sud-Est in the NTZ; IF: Ile aux Fous and RB: Rivière Banane in GPZs). At all sites, the abundance and taxonomic composition of coral recruits was characterised at 12 m depth on unglazed terracotta settlement tiles [64–68]. For each site, 20 individual tiles (*ca*. 10 × 10 × 2 cm) were immersed for six months over two austral summer periods (October 2015–March 2016, October 2016–March 2017) and one winter period (April–September 2016). Tiles were deployed and spatially referenced within each site at Reunion by measuring distances and azimuth between each tile and a reference point. Typical distance between two consecutive tiles was 1–2 m, while most distant tiles were around 15 to 20 m apart from each other. At the end of the immersion periods, the tiles were retrieved, bleached and sun dried to expose the skeleton of coral recruits (coral spats) for identification and counting under a dissecting microscope. Recruits of the families Acroporidae, Pocilloporidae and Poritidae were discriminated according to morphological traits [69]. Other recruits were assigned to the category ‘others’ if not associated with one of the previously cited families, or to the category ‘broken’ when the skeleton was too damaged for identification [34].

### Data analyses

In all the analyses described hereafter, abundance of recruits counted per tile was expressed as recruitment rate per m^2^ (recruitsm^–2^). Spatio-temporal variation of overall recruitment rates (all taxa combined) was evaluated at three different spatial scales: between islands, between protection levels (i.e. within NTZ or outside MPA), and between sites, for three periods (two summers and one winter) using an ANOVA model (Eq 1).

$$T_{ijkl}=m+I_{i}+Pr_{j}+S_{k}+{\mathrm{Pe}}_{l}+{\left(IPe\right)}_{il}+{\left(PrPe\right)}_{jl}+\varepsilon_{ijkl}$$ (Eq 1)

where *i*, *j*, *k* and *l* stand for the island, the protection levels, the sites and the periods, respectively, and ε is the residual variance (within sites). The spatial factors correspond to a three-level nested design with protection levels within islands and sites within protection levels. Sites were considered as a random factor. Prior to analyses, normality and homogeneity of the variance of the data set were tested using Shapiro-Wilk and Bartlett tests, respectively. Recruitment rate variable was log(x+1) transformed to meet the assumptions of normality. To analyse differences between levels of the significant factors, Student Pairwise t-tests (SPT) were used.

Spatio-temporal variation of the taxonomic composition was also evaluated at the three different spatial scales (between islands, between protection levels and between sites) for three periods using Chi-squared tests or Fisher’s exact test when assumptions for Chi-squared test were not met.

The spatial variation of coral recruitment between tiles was determined by evaluating the effect of geographical distance between pairs of tiles using a Mantel test. These tests will be referred to as Mantel 1 for overall recruitment rates and Mantel 2 for taxonomic composition. Temporal variations of overall recruitment rates were then tested using a Spearman rank correlation test to compare the overall recruitment rates between all period pairs, for each site.

Spatio-temporal variation of recruitment within tiles was assessed by comparing the orientation of recruits over the different surfaces of the tiles (upper, sides and lower) between islands, protection levels and periods independently for four taxonomic categories (Acroporidae, Pocilloporidae, Poritidae and other families) using Chi-squared tests or Fisher’s exact test when assumptions for Chi-squared test were not met.

## Results

### Spatio-temporal variations of recruitment rates

**Fig 2:**
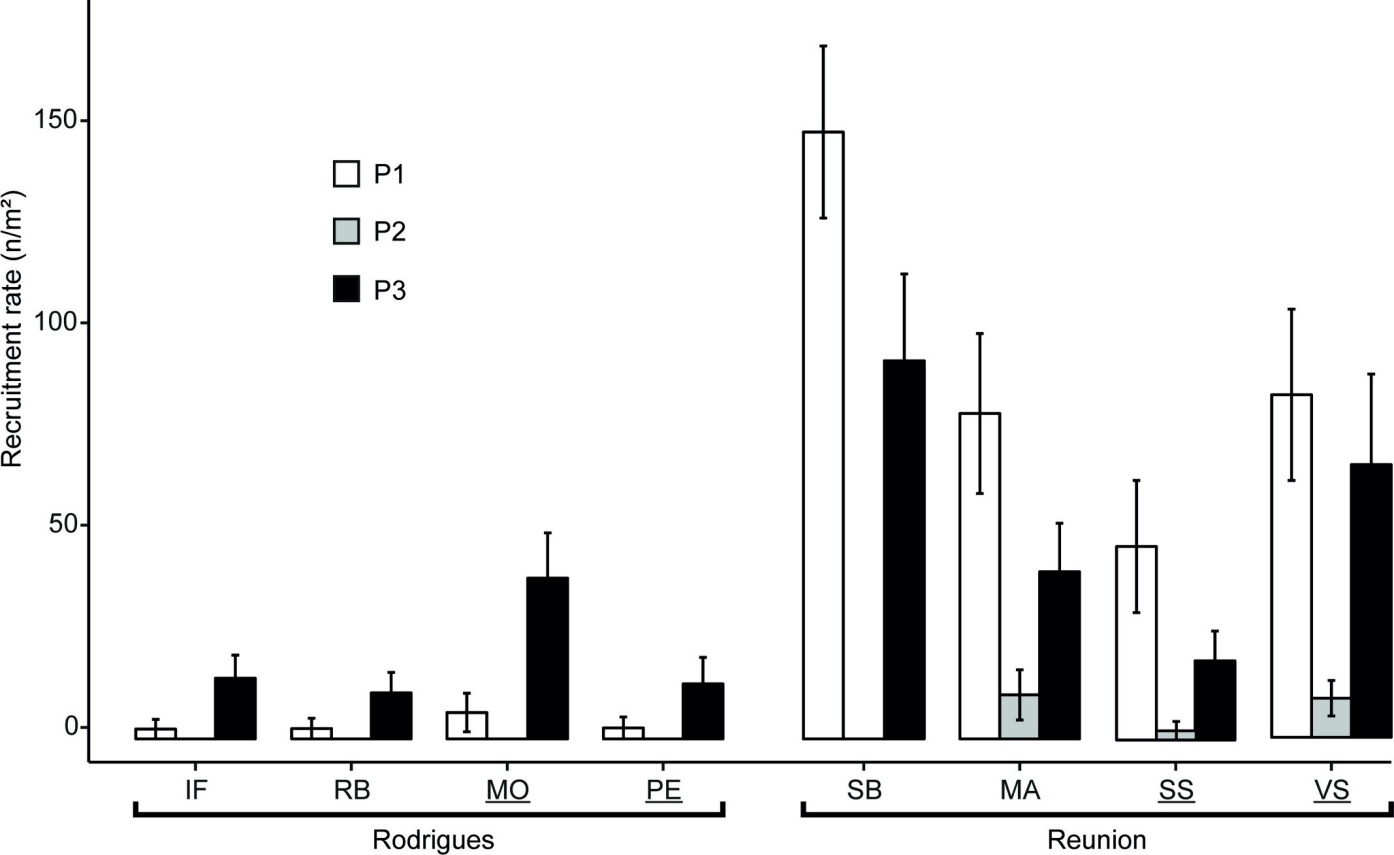
Recruitment rates observed during three consecutive 6-month periods on artificial settlement tiles at 12 m depth among reef slope sites on Rodrigues and Reunion reefs (recruitsm^–2^ ± SE). P1: period 1, i.e. summer 2015–2016 (October 2015–March 2016); P2: winter 2016 (April–September 2016); P3: summer 2016–2017 (October 2016–March 2017). NTZs are underlined. No recruits were observed at Rodrigues (at any site) or at SB site during winter 2016. SS: Sanctuaire Sud; SB: Souris Blanche; VS: Varangue Sud; MA: Marine; IF: Ile aux Fous; RB: Rivière Banane; MO: Mourouk; PE: Port Sud-Est.

Recruitment rates ranged from 48 to 150 recruitsm^–2^ during the first summer (October 2015–March 2016) at Reunion while fewer than 7 recruitsm^–2^ were recorded over the same period at Rodrigues (Fig 2). Over the following summer (October 2016–March 2017), recruitment rates were slightly lower at Reunion (20–98 recruitsm^–2^) compared to the previous summer, while they were higher than observed the year before at Rodrigues, reaching 11–40 recruitsm^–2^. During the winter (April–September 2016), recruitment rates were extremely low, with fewer than 11 recruitsm^–2^ recorded at Reunion and no recruits at all at Rodrigues. Recruitment rates (all taxa pooled) varied significantly between islands (ANOVA, *P* < 0.0001; Table 1) and among periods (ANOVA, *P* < 0.0001; Table 1). Moreover, the island × period interaction was significant (ANOVA, *P* < 0.0001; Table 1). These differences between periods were mainly due to recruitment variation between both summers and the winter (SPT, *P* < 0.0001; Fig 2), while there were no significant differences between the two summers (SPT, *P* > 0.05; Fig 2). Also, recruitment rates were slightly higher in the NTZ in Rodrigues compared to the GPZ, while it was the contrary at Reunion (Fig 2). Nevertheless, effects of protection level or protection × period interaction on recruitment rates were not significant (ANOVA, *P* > 0.05; Table 1).

**Table 1:** ANOVA table of the analyses of spatio-temporal variations of coral recruitment rates.

Factor	Df	Mean Square	F	P-value
Island	1	90.378	75.9253	2.20E-16
Protection (within Island)	2	5.153	4.3292	0.67
Site (within Protection)	4	5.864	4.926	5.92E-04
Period	2	48.318	40.5911	2.20E-16
Island x Period	2	26.175	21.9896	3.50E-10
Protection (within Island) x Period	4	2.046	1.719	0.14
Residuals	2204	1.19		

Df: degrees of freedom; F: F-statistic.

A significant inter-site variability of recruitment rates was recorded (ANOVA, *P* < 0.001; Table 1). While no differences were found between Rodrigues sites, post-hoc tests revealed significant differences between each pair of Reunion sites except between MA and VS, and MA and SS (SPT, 0.0001 < *P* < 0.05; Fig 2).

**Fig 3:**
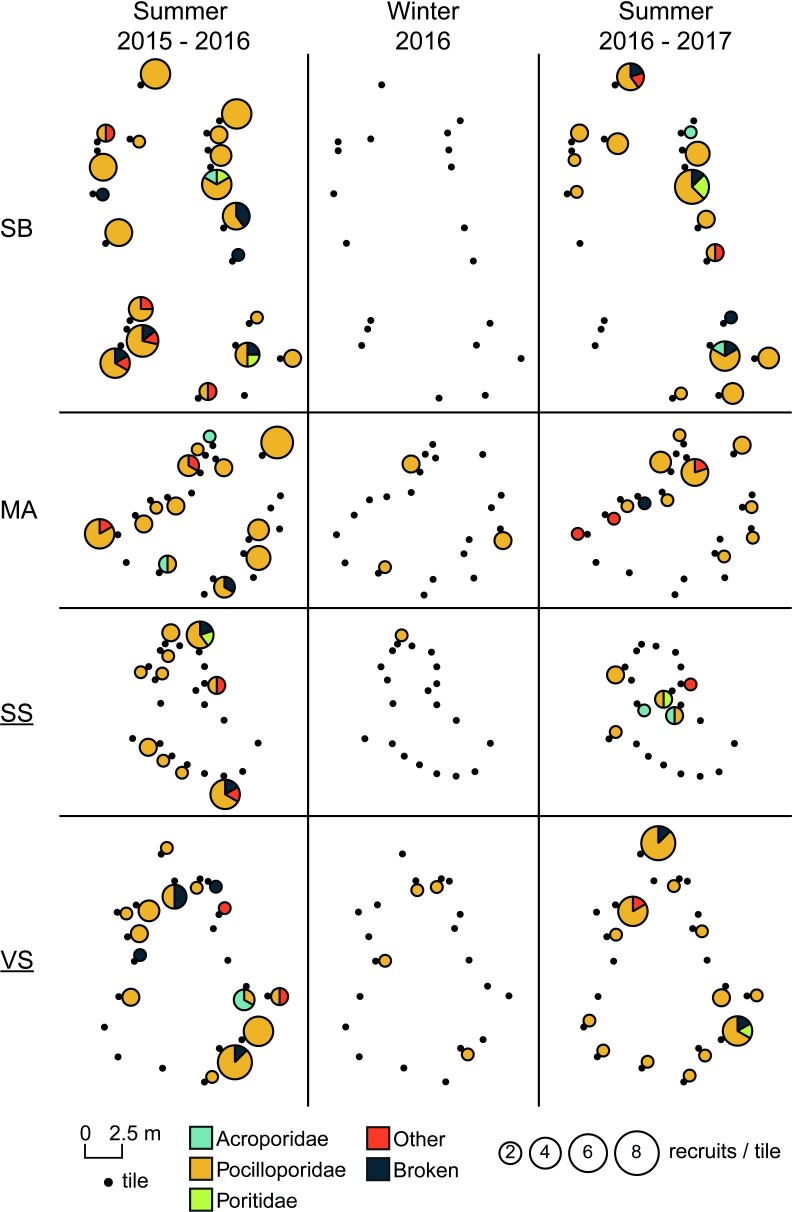
Spatio-temporal variations of number and composition of recruits observed on settlement tiles as they were arranged on the field. Data presented for the four sites at 12 m depth at Reunion, for the three studied periods. Size of pie chart is proportional to numbers of recruits. NTZs sites are underlined. SS: Sanctuaire Sud; SB: Souris Blanche; VS: Varangue Sud; MA: Marine.

Moreover, an important variability of recruitment rates among individual tiles was observed within each site at Reunion, whatever the protection level (Fig 3). However, no aggregative effects were highlighted among groups of tiles; the geographical distances between tiles were not related to recruitment rates observed on tiles (Mantel 1, *P* > 0.05; Fig 3). From a temporal point of view, there was no relationship in recruitment rates on individual tiles within each site between the two summers (2015–2016 and 2016–2017; Spearman rank tests, *P* > 0.05; Fig 3). This means highest recruitment rates were not observed on the same tile spots over the two consecutive summers.

### Spatio-temporal variations of taxonomic composition

**Fig 4:**
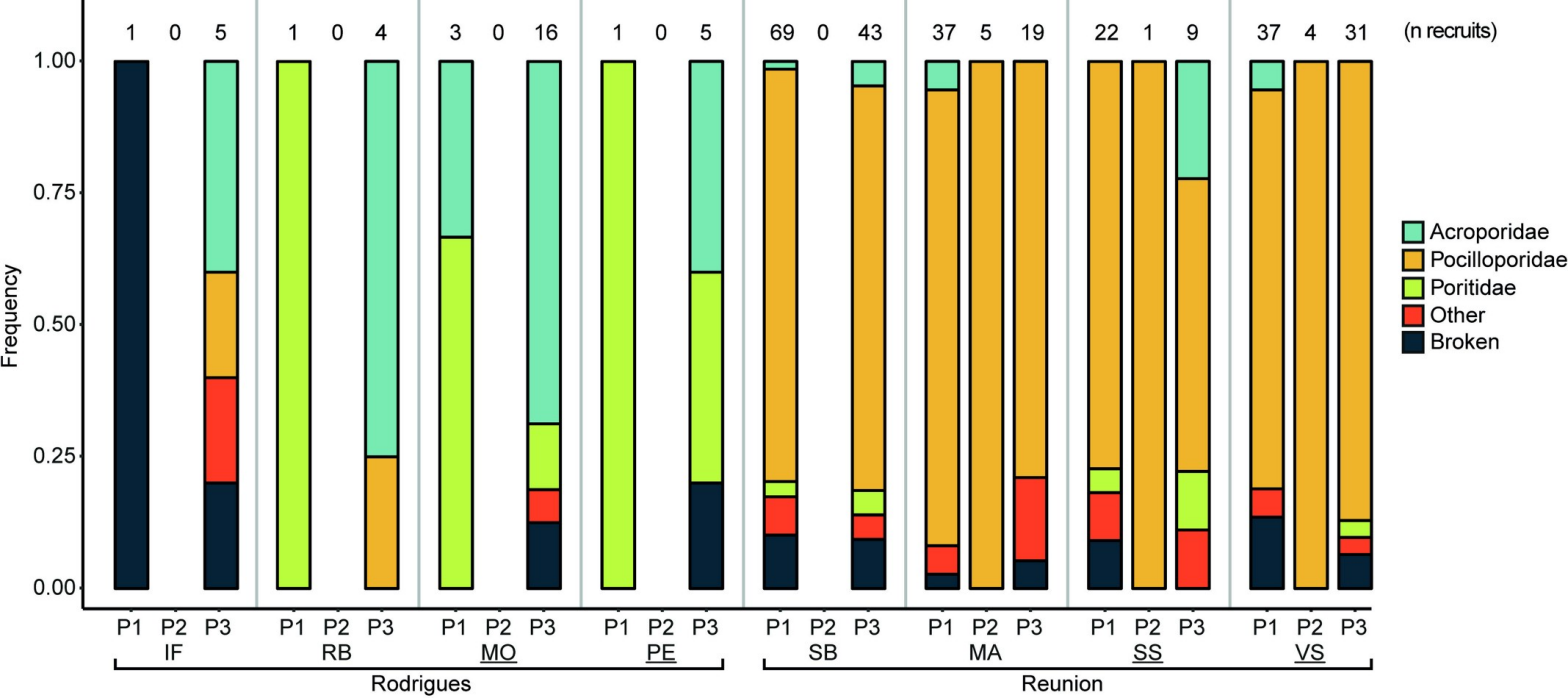
Taxonomic composition of coral recruits observed on artificial settlement tiles at 12 m depth among reef slope sites on Rodrigues and Reunion reefs. P1: period 1, i.e. summer 2015–2016 (October 2015–March 2016); P2: winter 2016 (April–September 2016); P3: summer 2016–2017 (October 2016–March 2017). NTZs sites are underlined. SS: Sanctuaire Sud; SB: Souris Blanche; VS: Varangue Sud; MA: Marine; IF: Ile aux Fous; RB: Rivière Banane; MO: Mourouk; PE: Port Sud-Est.

The relative contribution of the different families varied significantly between islands (Chi-squared test, *P* < 0.0001; Fig 4). During the two summers, a large dominance of Pocilloporidae (86% on average) was observed at Reunion (Fig 4). At Rodrigues, a shift of the dominant family between summers was noted. Poritidae recruits dominated the first summer (October 2015–March 2016, 80%) while Acroporidae recruits dominated the second summer (October 2016–March 2017; 69%). Consistently, taxonomic composition only varied significantly between the two summers at Rodrigues (Fisher’s exact test, *P* < 0.04; Fig 4) and not at Reunion (Fisher’s exact test, *P* > 0.05). During the winter, all recruits recorded at Reunion (n = 10) belonged to the Pocilloporidae family.

At both islands, taxonomic composition did not differ significantly between NTZs and GPZs (Fisher’s exact tests, *P* > 0.05; Fig 4). Moreover, no difference of taxonomic composition was observed for each pair of sites within both islands (Fisher’s exact tests, *P* > 0.05; Fig 4).

As with recruitment rates, taxonomic composition was not related to the relative position of tiles within sites (Mantel 2, *P* > 0.05; Fig 3), implying that tiles close to each other did not display more similar relative proportions of taxa than distant ones.

### Spatio-temporal variations of the orientation of recruits

Orientation of recruits on tiles varied significantly between islands (Fisher’s exact test, *P* < 0.01). At Rodrigues, recruits settled mainly on the sides of the tiles, to a lesser extent on the upper side and never on the lower surfaces where light availability was the lowest. At Reunion, recruitment was more evenly distributed, although recruitment rates were higher on the sides of the tiles than on other orientations. The orientation of recruits on tiles was not influenced by the protection level nor by the time period for both islands (Fisher’s exact tests, *P* > 0.05).

## Discussion

### Abundance of recruits

The present study is the first published on coral recruitment within the Mascarene Islands. Results reveal that overall recruitment rates measured on outer reef slopes at Reunion and Rodrigues islands from 2015 to 2017 were low, not exceeding 150 recruitsm^–2^ at Reunion and 40 recruitsm^–2^ at Rodrigues, during the summer. When compared to other reefs in the SWIO, recruitment rates from the present study were much lower than those on the Nyali reef in Kenya (908 recruitsm^–2^ y^-1^; [53]), along the coast of South Africa (1000 recruitsm^–2^ during peak settlement; [70]), or on the reefs of Vamizi island in Mozambique (1130 recruitsm^–2^ y^-1^; [10]). However, the recruitment rates at Reunion and Rodrigues islands were similar to those recorded at Coral Gardens in Kenya (101 recruitsm^–2^ y^-1^; [53]). Recruitment rates observed in this study were also comparable to levels observed in Moorea, French Polynesia [34,66,71] and in some locations of the Red Sea [8]. Even if artificial settlement tiles have been commonly used as a standardised assay of coral recruitment, the comparison of coral recruitment on artificial materials is challenging, due to varying methodology, as noted by Edmunds [72].

### Variations of recruitment through protection level

Contrary to what was expected, no significant differences in recruitment rates were found between the NTZs and GPZs. Preservation of fish stocks by NTZ implementation is known to foster herbivore communities and consequently to promote recovery potential and thus resilience of coral reefs [73–75]. Indeed, herbivory is one of the major factors controlling algal development, which inhibits coral recruitment through competition for space and smothering of recruits by trapped sediments [76]. However, herbivorous grazers, like fish and urchins, can also exert incidental predation on young coral stages [24,77–79]. Overall, most authors consider increasing herbivory as beneficial for coral recruitment (see reviews by [44,80]).

Lack of protection effect on coral recruitment rates in the studied locations may have several causes. The NTZs of both Reunion and Rodrigues islands were set up less than 10 years ago and the direct role of fishing pressure reduction on coral recruitment might not be effective yet. Absence of positive effects on the coral communities in recently protected areas has been previously highlighted [81,82]. In addition, Graham et al. [44] noted that spatial differences in recruitment rates may not necessarily align with NTZs placement and therefore, even if NTZs are effective at enhancing coral cover, this would not necessarily translate into higher local recruitment. These results support the idea that coral recruitment is sensitive mainly to large-scale disturbances whose deleterious effects cannot be directly mitigated by the establishment of a NTZ. In this context, it may be worthwhile to investigate the benefits of placing NTZs in areas of high recruitment, as they are more likely to recover from disturbances than areas with low recruitment rates.

### Spatial variability of recruitment

We found significant differences in recruitment rates among Reunion sites while both recruitment rates and taxonomic composition were similar in all sites at Rodrigues. This was surprising as sites at Reunion were all facing west, but sites at Rodrigues faced south-east and north. Thus, differences in recruitment rates could not be attributed to site configuration alone. At Rodrigues, the similarity in taxonomic composition may be mainly due to the low number of recruits.

Rodrigues is a very small and isolated island (about 600 km eastward from Mauritius and several thousand kilometres away from the land towards the other cardinal points). Reefs of the Mascarene Islands are under the influence of the South Equatorial Current (SEC) flowing east to west. Larval dispersal simulations highlighted connections within the Mascarene Islands from Rodrigues to Reunion [83,84]. This is consistent with some recent genetic studies conducted on *Pocillopora damicornis* type beta (Pocilloporidae) in the SWIO highlighting that unidirectional gene flows occur, even if limited, from east to west [85]. Moreover, Gélin et al. [86] showed that populations of *P*. *damicornis* type beta from the south of Reunion were genetically differentiated from those located in the north and the west of the island. The authors attributed this genetic isolation to a particular connection between the southern reefs of Reunion and Mauritius or Rodrigues reefs through the SEC. However, it was also suggested that the complex currents occurring along the west coast of Reunion might prevent non-local larvae from reaching the western reef, and instead only retain local larvae [86]. Thus, it could be interesting to (i) add sites on the south coast of Reunion to determine whether recruitment rates and taxonomic composition are closer to those observed at Rodrigues and (ii) to combine recruitment and genetic analyses to determine the origin of the recruits and to better understand gene flow occurring among the Mascarene Islands.

Among the different settlement tiles of each site, recruitment rates and taxonomic composition varied greatly, reflecting a patchy distribution, as previously observed in French Polynesia [34] and on the GBR [87]. This variability has also been observed in the structure of adult and juvenile corals in many reefs (e.g. [88,89]), and is likely to be related to the strong environmental heterogeneity encountered at small scales in reef ecosystems [90] and induced by different factors, such as local-scale hydrodynamic regimes [91], disturbance history [92] or predation [50]. However, no aggregative effect was revealed among spatial groups of tiles when tested within sites at Reunion. This means that tiles with high numbers of recruits were not located in the same area of the site, or particularly close to each other. In parallel, tiles close to each other did not display more similar relative proportions of taxa than distant ones. In terms of methodology, this shows that spatial variability at the scale of the site is correctly sampled. At the same spatial scale, highest recruitment rates were not observed on the same tile spots over the two consecutive summers, suggesting that processes responsible for spatial variation at this scale are not spatially consistent among years.

Within tiles, higher recruitment rates were found on the sides of the tiles deployed both at Reunion and Rodrigues islands, compared to the upper and lower surfaces. These observations were especially true at Rodrigues, where recruits rarely settled on the upper and lower faces of tiles. This may be linked to the higher concentration of suspended matter at Rodrigues than at Reunion (Jouval, pers. obs.). Indeed, this could (i) increase sedimentation on the upper surface of tiles, preventing the recruits from settling on this face and (ii) limit light penetration through the water column, which can prevent recruits from settling on the shady (lower) surface of the tiles. The effects of sedimentation and light availability on coral larvae settlement are often invoked as factors influencing the position of recruits over tiles [11,38,93]. The very weak presence of Pocilloporidae recruits on the upper faces of tiles, while being the most represented recruits in our study, illustrates their sensitivity to sedimentation, as previously observed [94]. The low proportion of recruits found on the upper surfaces of the tiles at both islands may also be a consequence of grazing by herbivorous fish and/or urchins [34,71]. Larval sensitivity to high-intensity light highlighted by other studies (e.g. [95,96]) is unlikely to have inhibited settlement of recruits on upper surfaces of the tiles in our study because of the depth of immersion of the tiles.

### Temporal variability of recruitment

At each spatial scale, from regional (between islands) to micro-local (within tiles), a strong period effect was highlighted both on recruitment rates and taxonomic composition, linked to the very low recruitment rates during the winter (April–September 2016), with only Pocilloporidae recruits at Reunion and the complete absence of recruits at Rodrigues. These results were consistent with observations made in the Indo-Pacific, where larval settlement is highly seasonal (e.g. reviewed in [97], [34] in French Polynesia, [98] on the Great Barrier Reef). The dominance of Pocilloporidae recruits on Reunion reefs was consistent with many observations across tropical and sub-tropical reefs of the Indo-Pacific [34,50,53,70,99]. The presence of some Pocilloporidae recruits during the winter at Reunion may have resulted from local asexual reproduction of adult colonies, already documented for this family, by polyp bail-out (*Seriatopora hystrix*, [100]) or by the production of parthenogenetic larvae (*Pocillopora damicornis*, [101]). In particular, at Reunion, Gélin et al. [86] suggested that the lagoonal species *P*. *damicornis* produces parthenogenetic larvae, but information is lacking for other Pocilloporidae species present on reef slopes. During the two summers (October to March), recruitment rates were higher and the taxonomic composition more diversified compared to the winter (April to September). On Rodrigues reef, recruits of Poritidae were dominant in the first summer, but Acroporidae recruits were highest in the second, while recruitment rates were slightly higher. The first summer studied corresponded to the massive bleaching event of 2016 that affected coral communities of Reunion and Rodrigues islands, thus potentially modifying coral recruitment rates and taxonomic composition. Indeed, numerous studies have revealed that adults of the Acroporidae family are among the most susceptible to bleaching events [102–104]. The 2016 coral bleaching greatly affected adults of Acroporidae species, especially *Acropora abrotanoides*, which was dominant in some sites at Rodrigues, leading to the death of the majority of them (Jouval, pers. obs.). Since recruitment at Rodrigues is likely to rely mainly on local coral populations, based on the observations about connectivity and currents, this could explain the greater representation of Poritidae recruits in the year of bleaching in Rodrigues. Nevertheless, these observations are related to an overall very low number of recruits at Rodrigues, and thus need to be considered with caution. On the tiles, the highest or lowest recruitment rates were not detected on the same tile from one period to the next. Spatial patterns of variation of recruitment rates within sites were not consistent over the three consecutive periods studied.

## Conclusions

Our results demonstrate that recruitment rates were low in the Mascarene Islands, especially at Rodrigues. Recruitment patterns varied greatly at several spatial scales: between islands, and between and within tiles. To a lesser extent, recruitment rates also varied between sites only at Reunion. Both recruitment rates and taxonomic composition displayed great seasonal variation, with very low recruitment rates during the winter. These observations suggested the importance of studying a minimum number of sites, each represented by several replicates (i.e. tiles), to correctly assess the spatio-temporal variability of recruitment patterns between or within reefs. The spatio-temporal variations of recruitment patterns described here have direct implications in terms of conservation, showing that some sites present higher overall recruitment rates, potentially giving these sites a higher capacity for recovery following disturbances. Our study did not highlight any effect of protection on coral recruitment. With low recruitment rates, versatile taxonomic composition over time, and high concentration of suspended matter, Rodrigues appears to be particularly vulnerable to large-scale disturbances.
